# Case Report: Pediatric Hallucinations and Anti-Neuronal Intermediate Filament Autoimmune Encephalitis

**DOI:** 10.5811/cpcem.48533

**Published:** 2025-01-01

**Authors:** Amanda H. Bjornstad, Natalie Oberhauser-Lim, Tammy Phan, Emmelyn J. Samones, Timothy Young

**Affiliations:** Loma Linda University Medical Center, Department of Emergency Medicine, Loma Linda, California

**Keywords:** Autoimmune encephalitis, pediatric, hallucinations, neuronal intermediate filament, case report

## Abstract

**Introduction:**

Patients with psychiatric concerns often present to an emergency department (ED) for medical evaluation prior to inpatient psychiatry placement. One diagnosis to consider prior to disposition is autoimmune encephalitis (AIE). This report describes a pediatric patient who presented with psychiatric symptoms that required inpatient admission and workup to diagnose a rare form of AIE.

**Case Report:**

A 16-year-old female with no known past medical history presented as a transfer from an outside hospital for medical evaluation of two days of auditory and visual hallucinations. Initial labs and imaging were unremarkable. Due to the acuity of her symptoms and abnormal vital signs, she was admitted to the hospital for further medical workup. After almost three weeks inpatient and multiple specialist consultations, she was diagnosed with anti-heavy chain neuronal intermediate filament AIE. The next month of admission included treatment with immunomodulators, antibiotics for associated infections, and malignancy evaluation. Symptoms resolved, and the patient was discharged. The patient remained asymptomatic on immunotherapies, and without psychiatric medications, the following year.

**Conclusion:**

During evaluation of psychiatric concerns in the ED, it is essential to consider organic causes of behavioral changes, which can be difficult to discern. Autoimmune encephalitis can be subtle. Features such as autonomic dysregulation, acute or subacute symptom onset, recent infection, autoimmune or malignancy history, cognitive deficits, or focal neurologic findings should raise clinical suspicion. For patients with psychiatric symptoms, the role of an emergency physician is not to diagnose autoimmune encephalitis, but to recognize nuances in patient presentations to best direct proper workup, treatment, and disposition.

## INTRODUCTION

Mental health concerns are common in pediatric emergency departments (PED).^1^ Often, children are sent to PEDs for medical stabilization prior to admission to inpatient psychiatric facilities. One uncommon, but vital, diagnosis to consider prior to disposition is autoimmune encephalitis (AIE). Autoimmune encephalitis is a central nervous system (CNS) inflammatory disease process caused by an autoimmune response toward various CNS antigens.^2^–^4^ Presenting symptoms are vast and variable, including gastrointestinal (GI) upset, headache, seizures, movement disorders, and behavioral changes that can resemble psychosis.^2^,^3^,^5^

If left unrecognized or untreated, AIE can have devastating effects, including persistent cognitive dysfunction, neurologic deficits, and death, with mortality rates as high as 6%.^2^ Although AIE is uncommon, with an incidence of five to eight cases per 100,000 people in the general population, prompt identification, workup, and disposition are essential, as early diagnosis and treatment have been shown to significantly improve outcomes.^2^–^4^ Emergency physicians will frequently be the first to recognize symptoms and determine which patient presentations require further investigation.

## CASE REPORT

A 16-year-old, fully vaccinated female in eleventh grade with no past medical history presented as a transfer from an outside hospital for acute onset auditory and visual hallucinations. The patient endorsed hallucinations that started in seventh grade; contrary to this, her parents reported no psychotic symptoms prior to the 48 hours before presentation. The patient reported auditory and visual hallucinations of a flying green dinosaur, hearing voices that told her to hurt herself, and a sense that people were following her. Throughout the interview, the patient responded to internal stimuli and reacted to her visual hallucinations of the dinosaur.

Per the patient’s mother and father, within the prior month the patient’s excellent grades declined, and her interest in social interaction and participation in extracurricular activities diminished. Her parents brought her to the PED due to worsening bizarre behavior and actively interacting with hallucinations for two days, which they had not previously observed. The patient and her family traveled to the Philippines three weeks prior, and multiple members of the family, including the patient, developed a cough during this time. Per parents, the patient did not have a history of substance use, emotional or physical trauma, psychiatric diagnoses, or need for psychotherapy. Additionally, there was no family history of schizophrenia or other psychiatric disorders.

Upon presentation to the PED, the patient was afebrile, normotensive, persistently tachycardic to the 140s beats per minute, tachypneic to the 20s breaths per minute but saturating well on room air. Of note, she was febrile to 100.6º Fahrenheit at the transferring hospital. Physical exam was notable for tremulousness, diaphoresis, no respiratory distress or abnormal lung sounds, tracking and interacting with internal stimuli, normal neck range of motion, normal pupils without significant miosis or mydriasis, and non-focal neurologic exam, including normal gait. The patient’s abnormal vital signs, ill appearance, and rapid symptom onset raised concern for organic etiology.

Initial workup at the transferring hospital had the following pertinent findings: no leukopenia/leukocytosis; no anemia; no thrombocytopenia/thrombocytosis; no electrolyte derangements; normal thyroid-stimulating hormone; negative urine drug screen; urinalysis with no infection, hematuria, proteinuria, or glucosuria; and chest radiograph with no abnormalities. In the PED, a non-contrast computed tomography (CT) head was unremarkable with no mass, edema, or hemorrhage. Lumbar puncture was attempted in the PED without return of cerebrospinal fluid (CSF). No antibiotics were started in the PED due to low suspicion for meningitis given the absence of meningismus, lethargy, seizures, or significant lab or CT findings. However, due to her persistent ill appearance and vital sign abnormalities, the PED team determined she was not medically stable for psychiatric facility admission; therefore, the general pediatric team was consulted for admission.

*CPC-EM Capsule*

What do we already know about this clinical entity?

*Patients with psychiatric concerns commonly present to the emergency department (ED) for medical evaluation; underlying organic causes can be difficult to identify.*

What makes this presentation of disease reportable?

*This patient presented with a common pediatric ED concern of hallucinations but with signs of autoimmune encephalitis (AIE).*

What is the major learning point?

*History and exam findings that favor AIE versus psychiatric diagnosis include acute or subacute presentation, recent infection, and history of autoimmunity.*

How might this improve emergency medicine practice?

*Enhanced clinical suspicion for AIE in patients with psychiatric concerns could improve patient outcomes.*

Upon admission, an electroencephalogram was completed, per the recommendation of the pediatric neurology team, with no abnormal findings. A rapid response was called on admission day two due to the patient demonstrating complete catatonia, for which the consulting psychiatry team recommended treatment with lorazepam; catatonic symptoms subsequently improved over a period of days. The psychiatric team’s initial differential diagnosis included psychosis but emphasized the need to rule out organic etiology. Additional unremarkable workup included magnetic resonance imaging (MRI) of the brain with and without contrast, respiratory viral panel, pregnancy test, human immunodeficiency virus (HIV) testing, hepatitis panel, and herpes simplex virus types 1 and 2 serum polymerase chain reaction.

A lumbar puncture was performed by the interventional radiology team, and the following CSF studies were normal: cell count; glucose; protein; meningitis panel (viral, bacterial, and fungal); and culture. On hospital day 4, a GI panel resulted positive for cryptosporidium, for which she was treated with nitazoxanide. On hospital day 8, the consulting psychiatry team recommended inpatient psychiatry transfer. The patient was deemed medically stable by the primary pediatrics team, but she developed difficulty with independent feeding that precluded transfer. Serum and CSF autoimmune encephalitis panels were pending at this time. Per the recommendation of the consulting psychiatry team, olanzapine was added to her medication regimen on hospital day 11 for treatment of persistent psychosis.

On hospital day 18, a CSF autoimmune encephalitis panel showed positive cell-based assay and titer (1:16) for anti-heavy chain neuronal intermediate filament antibody (anti-NIF Ab). Given the strong association of anti-NIF Ab with paraneoplastic processes, MRI of the chest, abdomen, and pelvis were completed but showed no evidence of malignancy. She ultimately received intravenous (IV) steroids, IV immunoglobulin (IVIG), rituximab, and five sessions of plasmapheresis over the next month. Due to the association between ehrlichiosis and anti-NIF Ab, she was empirically treated with doxycycline. On hospital day 57, the patient was discharged home on prednisone, olanzapine, lorazepam, and monthly IVIG. Outpatient positron emission tomography showed no evidence of neuroendocrine tumor or other malignancy. The patient now follows in outpatient clinic with reported continued resolution of symptoms more than one year after index presentation, continued immunomodulation therapy, and discontinuation of psychiatric medications.

## DISCUSSION

Autoimmune encephalitis can be difficult to diagnose due to its rarity. Also, much of pediatric AIE data is restricted to anti-N-methyl-d-aspartate receptor Ab (anti-NMDAR Ab), as it is the most commonly isolated auto-antibody and best characterized AIE syndrome in the pediatric population.^2^–^4^ However, many additional auto-antibodies have been identified as causes of pediatric AIE.^2^,^3^,^5^ Despite the uniqueness of pediatric anti-NIF autoimmune encephalitis, the post-infectious presentation is similar to many other pediatric AIE cases.

Infections documented to precede AIE include herpes simplex virus (strongly associated with anti-NMDAR autoimmune encephalitis), *Haemophilus influenzae*, enterovirus, mycoplasma, streptococcus, varicella zoster, cytomegalovirus, Ebstein-Barr, adenovirus, and rickettsial pathogens.^2^,^3^,^5^ Cases of post-infectious adult anti-NIF autoimmune encephalitis include anaplasma, HIV, ehrlichiosis, and severe acute respiratory syndrome coronavirus 2.^6^–^8^ Post-infectious anti-NIF autoimmune encephalitis is less common than paraneoplastic anti-NIF autoimmune encephalitis.^7^

Recognizing AIE, especially in an acute setting, is challenging due to symptom variability and subtlety. Common pediatric symptoms include autonomic dysfunction, movement disorders (eg, dystonia), language disorders (eg, mutism), sleep/wake cycle disturbances, and neurologic dysfunction (eg, seizures). Only 60% of pediatric patients present with psychiatric symptoms.^2^–^5^ This is different from adults who present with neuropsychiatric symptoms as a defining AIE feature.^4^ Cognitive dysfunction (eg, memory loss, inattention, etc.) and viral prodrome (eg, fever, headache, etc.) commonly occur in both populations.^4^,^9^,^10^ Anti-neuronal intermediate filament AIE, specifically, has three distinct phenotypes in adults: encephalopathy predominate; cerebellar-ataxia predominant; and myeloradicular neuropathies.^7^

In patients with psychiatric symptoms, differentiating psychological etiology from organic causes is clinically complex, and the two diagnoses may not be mutually exclusive. Psychiatric patients tend to have more anxious symptoms than AIE patients, although this may be difficult to clinically discern. “Red” and “yellow” flag symptoms that should raise clinical suspicion for AIE in psychiatric presentations are epileptiform activity, facial dystonia, bulbar symptoms, focal neurologic deficits, autonomic dysfunction, hyponatremia, headache, history of autoimmune disorders, and rapid psychosis progression despite treatment.^11^,^12^ Acute or subacute presentation may also be more suggestive of organic vs psychiatric etiology.^13^,^14^ In this particular presentation, the patient’s sustained tachycardia, diaphoresis (autonomic dysregulation), and symptom acuity raised suspicion for organic etiology.

A diagnostic approach exists for adult AIE, and although validated through some studies, there is opportunity to broaden inclusion criteria given significant variability in patient presentation.^14^,^15^ The current diagnostic criteria are considered highly sensitive and specific in pediatric populations, but most patients do not fulfill criteria until two weeks after symptom onset.^11^ Cellucci and colleagues have proposed modified pediatric criteria and an algorithm to account for differences from adult presentations.^3^ For an emergency physician evaluating a potential AIE patient, the proposed adult and pediatric criteria are beneficial to consider; however, it is essential to remember that AIE is not ruled out if criteria are not fulfilled completely. Diagnosis of AIE requires clinical suspicion, concordant history, extensive workup, and consideration of the most up-to-date diagnostic criteria and research

## CONCLUSION

Autoimmune encephalitis is rare and can be difficult to recognize. When determining whether to pursue an AIE workup, important historical data to acquire include the following: acuity of behavioral changes; recent infection; recent travel; personal or family history of autoimmune disorders and/or malignancy; prior psychiatric history; toxic ingestions; sleep cycle changes; and neurologic symptoms. Physical exam should include multiple sets of vitals to evaluate for autonomic dysregulation, thorough neurologic exam, and cognitive testing. Current AIE criteria and algorithms can also be helpful to support proper management but should not be used to rule out an AIE diagnosis. It is important to understand that a final diagnosis of AIE is not required in the emergency department, but it is a crucial diagnosis to consider and appropriately work up for safe disposition and treatment to improve patient outcomes.
